# Guidewire malposition outside the bile duct during endoscopic ultrasound-guided hepaticogastrostomy

**DOI:** 10.1055/a-2113-9777

**Published:** 2023-07-13

**Authors:** Yoshimitsu Fukasawa, Mitsuharu Fukasawa, Shinichi Takano, Satoshi Kawakami, Hiroshi Hayakawa, Shota Harai, Nobuyuki Enomoto

**Affiliations:** Department of Gastroenterology, Faculty of Medicine, University of Yamanashi, Yamanashi, Japan

Endoscopic ultrasound-guided hepaticogastrostomy (EUS-HGS) is widely used in clinical practice for patients with a malignant biliary obstruction after failed endoscopic retrograde cholangiopancreatography (ERCP) 1 2 . However, the rate of procedure-related adverse events associated with EUS-HGS is relatively high 1 2 . Herein, we report a case of guidewire malposition during EUS-HGS ( Video 1 ).

**Video 1** Guidewire malposition outside the bile duct during endoscopic ultrasound-guided hepaticogastrostomy.


An 80-year-old man with biliary obstruction due to pancreatic cancer ( Fig. 1 ) was referred to our hospital for EUS-HGS because of prior ERCP failure. During the procedure, the bile duct of segment 3 (B3) was punctured using a 19-G needle (EZ Shot 3 Plus; Olympus Medical, Tokyo, Japan), and contrast medium was injected into the bile duct. A guidewire (VisiGlide 2 Guidewires; Olympus Medical) was advanced toward the biliary tract without resistance ( Fig. 2 a ). After dilation of the fistula, contrast was noted outside the bile duct, suggesting guidewire malposition ( Fig. 2 b ).

**Fig. 1:**
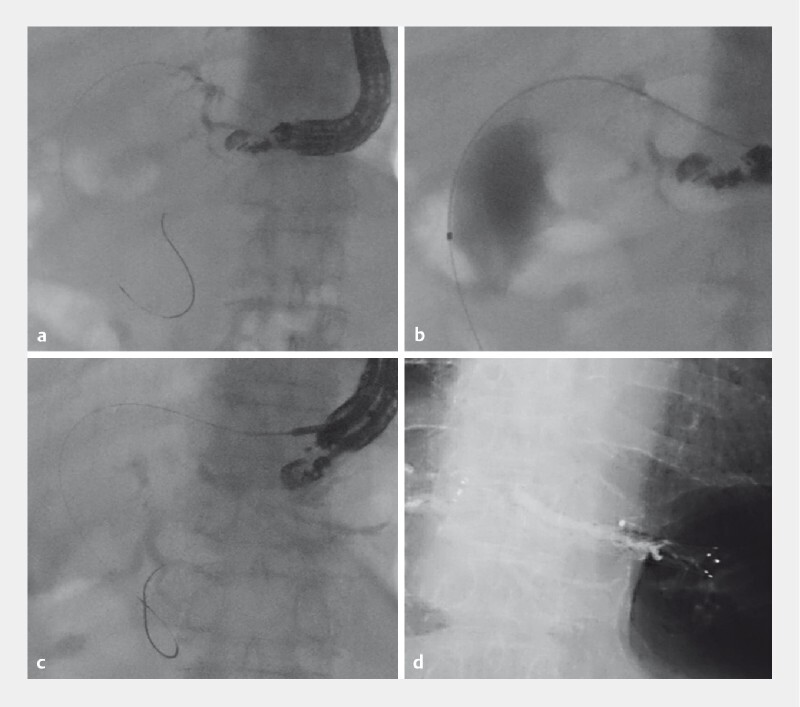
Preprocedure coronal computed tomography showing a dilated bile duct due to pancreatic head cancer (arrowhead).

**Fig. 2:**
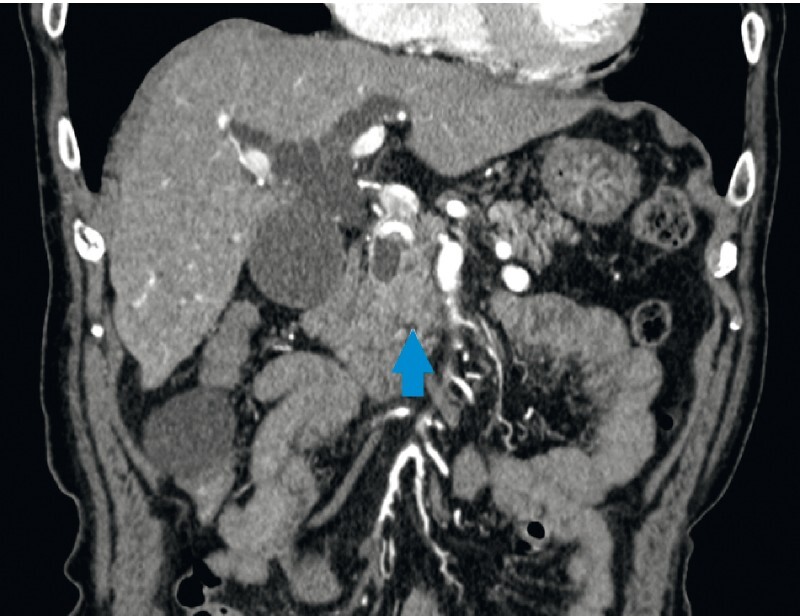
Fluoroscopic images from endoscopic ultrasound-guided hepaticogastrostomy (EUS-HGS) performed for biliary obstruction in an 80-year-old man with pancreatic head cancer. **a** In the first procedure, the bile duct of segment 3 (B3) was punctured and a guidewire was advanced toward the biliary tract. **b** Guidewire malposition was identified based on contrast extravasation outside the bile duct. **c** For salvage drainage, the bile duct of segment 2 (B2) was punctured and a guidewire was inserted into the biliary tract. **d** A self-expandable metal stent was placed during the EUS-HGS.

EUS-HGS on the bile duct of segment 2 (B2) was performed for salvage drainage. After the guidewire was inserted into the biliary tract ( Fig. 2 c ) and fistula dilation was performed, a fully covered self-expandable metal stent (X-Suit NIR Biliary Metallic Stents; Olympus Medical) was inserted ( Fig. 2 d ). Although the post-EUS-HGS computed tomography showed hematomas around the liver and spleen ( Fig. 3 ), the patient was discharged from our hospital after conservative treatment.

**Fig. 3:**
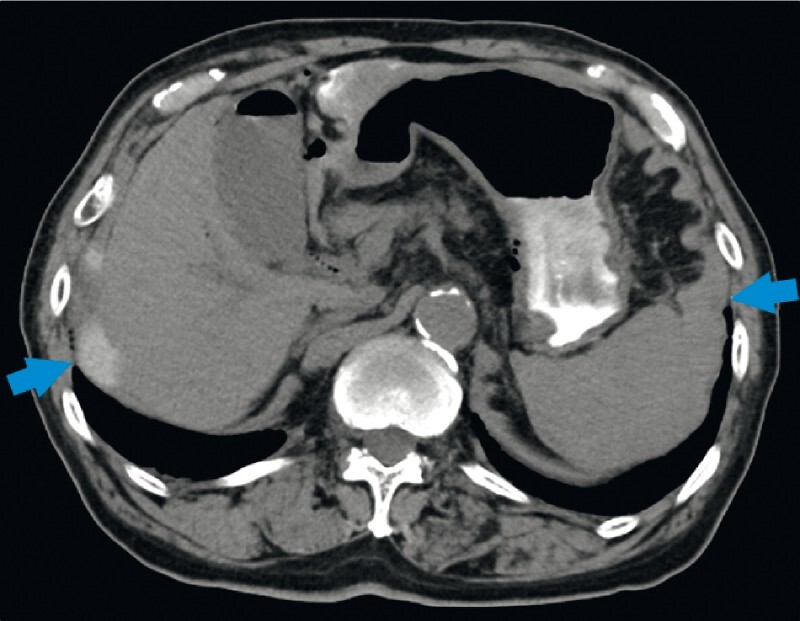
Postprocedure axial computed tomography showing hematomas around the liver and spleen (arrowheads).

The probable cause of incorrect advancement of the guidewire was incomplete placement of the needle tip within the bile duct. As a result, although the contrast medium was injected into the bile duct, the guidewire migrated outside the bile duct ( Fig. 4 ). Fluoroscopy showed that the two guidewires had a similar shape; however, their loop widths were different ( Fig. 5 a, b ). Recent advances have enabled EUS-HGS to be performed without fistula dilation, using a self-expandable metal stent with a small-diameter delivery system 3 4 . It is necessary to evaluate the shape of the guidewire before stent placement during EUS-HGS without fistula dilation.

**Fig. 4:**
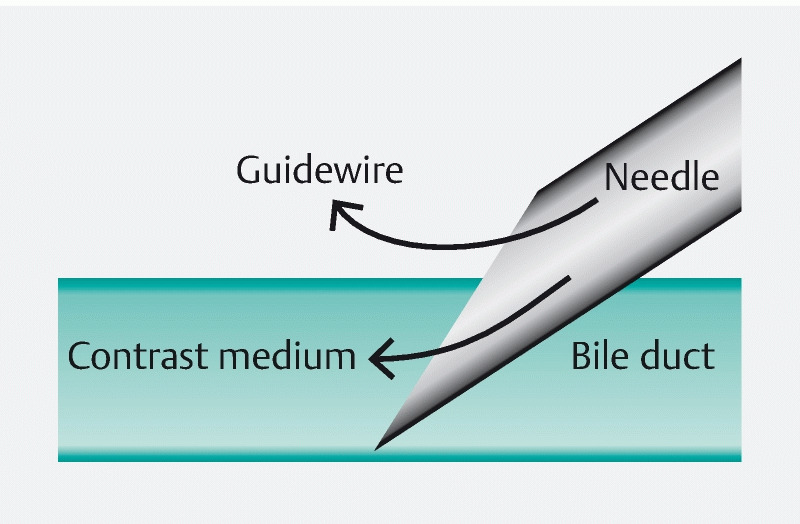
Relationship between the needle and bile duct in guidewire malposition. The needle tip is not completely inside the bile duct. As a result, although the contrast medium was injected into the bile duct, the guidewire migrated outside the bile duct.

**Fig. 5:**
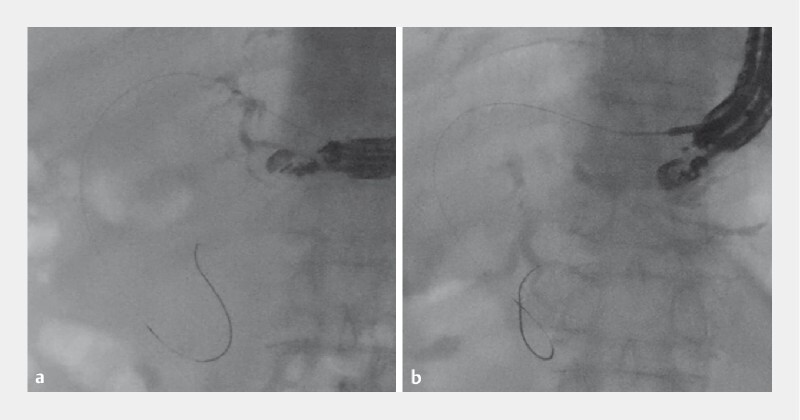
Fluoroscopic images showing the shape of the guidewire. Although the two guidewires had a similar shape, their loop widths were different. **a** Guidewire malposition outside the bile duct. **b** Correct insertion of the guidewire into the common bile duct.

Endoscopy_UCTN_Code_CPL_1AL_2AD

## Notes

Endoscopy E-Videos

https://eref.thieme.de/e-videos
*E-Videos* is an open access online section of the journal *Endoscopy* , reporting on interesting cases and new techniques in gastroenterological endoscopy. All papers include a high-quality video and are published with a Creative Commons CC-BY license. Endoscopy E-Videos qualify for HINARI discounts and waivers and eligibility is automatically checked during the submission process. We grant 100% waivers to articles whose corresponding authors are based in Group A countries and 50% waivers to those who are based in Group B countries as classified by Research4Life (see: https://www.research4life.org/access/eligibility/ ). This section has its own submission website at https://mc.manuscriptcentral.com/e-videos

## References

[1] Ogura T Higuchi K Technical review of developments in endoscopic ultrasound- guided hepaticogastrostomy Clin Endosc 2021 54 651 659 33896154 10.5946/ce.2021.020-KDDW PMC8505184

[2] Cho J H Park S W Kim E J Long-term outcomes and predictors of adverse events of EUS-guided hepatico-gastrostomy for malignant biliary obstruction: multicenter, retrospective study Surg Endosc 2022 36 8950 8958 35680668 10.1007/s00464-022-09346-z

[3] Ogura T Ueno S Okubo A Technical feasibility and safety of one‑step deployment of EUS‑guided hepaticogastrostomy using an 8‑mm diameter metal stent with a fine‑gauge stent delivery system (with video) Endosc Ultrasound 2021 10 355 360 34427190 10.4103/EUS-D-20-00206 PMC8544008

[4] Maehara K Hijioka S Nagashio Y Endoscopic ultrasound-guided hepaticogastrostomy or hepaticojejunostomy without dilation using a stent with a thinner delivery system Endosc Int Open 2020 8 E1034 E1038 32743055 10.1055/a-1169-3749 PMC7373653

